# Analysis of metabolic disturbances attributable to sepsis-induced myocardial dysfunction using metabolomics and transcriptomics techniques

**DOI:** 10.3389/fmolb.2022.967397

**Published:** 2022-08-15

**Authors:** Xiaonan Jia, Yahui Peng, Xiaohui Ma, Xiaowei Liu, Kaijiang Yu, Changsong Wang

**Affiliations:** ^1^ Departments of Critical Care Medicine, The First Affiliated Hospital of Harbin Medical University, Harbin Medical University, Harbin, China; ^2^ Departments of Critical Care Medicine, The Fourth Affiliated Hospital of Harbin Medical University, Harbin Medical University, Harbin, China; ^3^ Departments of Critical Care Medicine, Harbin Medical University Cancer Hospital, Harbin Medical University, Harbin, China

**Keywords:** sepsis, metabolic, myocardial dysfunction, SIMD, transcriptomics

## Abstract

**Background:** Sepsis-induced myocardial dysfunction (SIMD) is the most common and severe sepsis-related organ dysfunction. We aimed to investigate the metabolic changes occurring in the hearts of patients suffering from SIMD.

**Methods:** An animal SIMD model was constructed by injecting lipopolysaccharide (LPS) into mice intraperitoneally. Metabolites and transcripts present in the cardiac tissues of mice in the experimental and control groups were extracted, and the samples were studied following the untargeted metabolomics–transcriptomics high-throughput sequencing method. SIMD-related metabolites were screened following univariate and multi-dimensional analyses methods. Additionally, differential analysis of gene expression was performed using the DESeq package. Finally, metabolites and their associated transcripts were mapped to the relevant metabolic pathways after extracting transcripts corresponding to relevant enzymes. The process was conducted based on the metabolite information present in the Kyoto Encyclopedia of Genes and Genomes (KEGG) database.

**Results:** One hundred and eighteen significant differentially expressed metabolites (DEMs) (58 under the cationic mode and 60 under the anionic mode) were identified by studying the SIMD and control groups. Additionally, 3,081 significantly differentially expressed genes (DEGs) (1,364 were down-regulated and 1717 were up-regulated DEGs) were identified in the transcriptomes. The comparison was made between the two groups. The metabolomics–transcriptomics combination analysis of metabolites and their associated transcripts helped identify five metabolites (d-mannose, d-glucosamine 6-phosphate, maltose, alpha-linolenic acid, and adenosine 5′-diphosphate). Moreover, irregular and unusual events were observed during the processes of mannose metabolism, amino sugar metabolism, starch metabolism, unsaturated fatty acid biosynthesis, platelet activation, and purine metabolism. The AMP-activated protein kinase (AMPK) signaling pathways were also accompanied by aberrant events.

**Conclusion:** Severe metabolic disturbances occur in the cardiac tissues of model mice with SIMD. This can potentially help in developing the SIMD treatment methods.

## 1 Introduction

Sepsis is defined as a life-threatening organ dysfunction that is caused by an overreaction of the body to infection (Singer et al., 2016). It is a serious global problem and the most common cause of in-hospital mortality (Rudd et al., 2020). Sepsis-induced myocardial dysfunction (SIMD) is the most common and severe sepsis-related organ dysfunction. SIMD induces or exacerbates dysfunction in other organs. The prognosis of patients with SIMD is poor, resulting in an extremely high mortality rate (70–90%) (Martin et al., 2019; Ravikumar et al., 2021). It is known that the mechanisms underlying SIMD involve the release of circulating myocardial inhibitory substances, the release of nitric oxide and reactive oxygen species, abnormalities in calcium handling, downregulation of adrenergic pathways, and mitochondrial dysfunction (Hollenberg and Singer, 2021; Yang and Zhang, 2021). However, these abnormalities fail to explain the mechanisms underlying the onset and progression of SIMD. Circulating troponin and NT-proBNP exhibit good specificity and sensitivity in the cases of myocardial ischemic disease and cardiac failure. However, similar roles are not observed in the case of SIMD (Hollenberg and Singer, 2021). At present, a viable biomarker for SIMD is yet to be identified.

Metabolomics allows the exploration of small molecule metabolites in blood or tissues. Results obtained by conducting qualitative and quantitative analyses revealed that the relationship between metabolites and physiological/pathological changed over time (Rochfort, 2005; Patti et al., 2012). Metabolites are the end products of the biochemical activities occurring in the body. Therefore, metabolomics is the omics study that is closest to phenotyping. A number of metabolomics-oriented studies have been conducted to understand the pathogenesis, progression, and patient prognosis of sepsis (Neugebauer et al., 2016; Ping et al., 2019; Ping et al., 2021). However, there is a lack of such studies on SIMD. Additionally, transcriptomics facilitates the investigation of gene function and gene structure at a global level to identify differentially expressed genes (DEGs) within cells, tissues, or individuals under different physiological or pathological states (Velculescu et al., 1995; Virlon et al., 1999; Morris, 2009).

However, metabolomics solely may lead to incomplete findings. Therefore, metabolomics–transcriptomics combination analysis can be performed to accurately identify key metabolites, hub genes, and metabolic pathways associated with the ‘cause’ and ‘result’ dimensions (Rochfort, 2005; Griffin, 2006).

Mannose metabolism, amino sugar metabolism, starch metabolism, unsaturated fatty acid biosynthesis, platelet activation, and purine metabolism have some studies in sepsis (Gao et al., 2015; Bakalov et al., 2016; Hwang et al., 2019; She et al., 2022). However, there are no relevant studies in Sepsis-induced myocardial dysfunction (SIMD). The AMPK signaling pathway has some studies in SIMD (Song et al., 2020; Wang et al., 2021).

We aimed to investigate metabolite changes occurring in the heart tissues of mice suffering from SIMD. The SIMD-related metabolites and metabolic pathways were identified and studied by conducting untargeted metabolomics–transcriptomics combination analysis. Overall, the findings of this study provide new insights into the processes associated with the pathogenesis, early diagnosis, and treatment of SIMD.

## 2 Methods

### 2.1 Animal model establishment

Male C57BL/6 mice (age: 6–8 weeks) were purchased from Charles River (Beijing, China). The mice under study had free access to food and water. The mice belonging to the experimental group were administered intraperitoneal injections of lipopolysaccharide (LPS) (20 mg/kg) once to induce SIMD. The volume of saline that was administered intraperitoneally to the mice belonging to the control group was the same as the volume of LPS injections. The mice were subjected to conditions of echocardiography after 6 hours of injection, and 2D and M-mode echocardiographic measurements were taken under these conditions. A high-resolution *in vivo* imaging system (VIVID E9, GE, United States) was used to record the data. The left ventricular ejection fraction (LVEF), left ventricular end-diastolic dimension (LVEDd), left ventricular end-systolic dimension (LVESd), and left ventricular fractional shortening (LVFS) functioned as the measurement indicators. A short-axis view of the heart was obtained from the parasternal approach. The ejection fraction was also calculated. The formula for calculation is as follows:

$$\left(\mathrm{LVED}d^{3}-\mathrm{LVES}d^{3}\right)/\mathrm{LVED}d^{3}\times 100$$

The ejection fractional shortening was calculated as follows:

(LVEDd−LVESDd)/LVEd×100

Subsequently, the whole heart tissue samples were harvested following the execution of the mice. The blood in the heart chamber was rinsed with PBS. The samples were stored at a temperature of –80°C for subsequent use. All necessary permissions were obtained from the Ethics Committee of Harbin Medical University, and all procedures met the relevant regulatory standards.

### 2.2 Enzyme-linked immunosorbent assay

Commercially available enzyme-linked immunosorbent assay (ELISA) kits (Meimian Biotechnology, Jiangsu, China) were used to determine the levels of Tn-I. The instructions provided by the manufacturer were followed to conduct the studies.

### 2.3 Untargeted metabolomics studies

Heart tissue samples collected from mice belonging to the experimental group (*n* = 6) and the control group (*n* = 6) were slowly thawed at 4°C. Following this, the samples were treated with a pre-chilled solution consisting of methanol, water, and acetonitrile (methanol:acetonitrile:water = 2:2:1, v/v). The solution was vortexed, after which it was sonicated over a period of 30 min at a low temperature. Subsequently, the sample solution was allowed to stand at –20°C for 10 min, following which it was centrifuged at 14,000 g over a period of 20 min at 4°C. Subsequently, the supernatant was extracted, and it was dried under conditions of vacuum. The samples were analyzed using the mass spectrometry technique.

Analyses were performed using an UHPLC (1,290 Infinity LC, Agilent Technologies) coupled to a quadrupole time-of-flight (AB Sciex TripleTOF 6,600). For HILIC separation, samples were analyzed using a 2.1 mm × 100 mm ACQUIY UPLC BEH 1.7 µm column (waters, Ireland). In both ESI positive and negative modes, the mobile phase contained A = 25 mM ammonium acetate and 25 mM ammonium hydroxide in water and B = acetonitrile. The gradient was 85% B for 1 min and was linearly reduced to 65% in 11 min, and then was reduced to 40% in 0.1 min and kept for 4 min, and then increased to 85% in 0.1 min, with a 5 min re-equilibration period employed. For RPLC separation, a 2.1 mm × 100 mm ACQUIY UPLC HSS T3 1.8 µm column (waters, Ireland) was used. In ESI positive mode, the mobile phase contained A = water with 0.1% formic acid and B = acetonitrile with 0.1% formic acid; and in ESI negative mode, the mobile phase contained A = 0.5 mM ammonium fluoride in water and B = acetonitrile. The gradient was 1%B for 1.5 min and was linearly increased to 99% in 11.5 min and kept for 3.5 min. Then it was reduced to 1% in 0.1 min and a 3.4 min of re-equilibration period was employed. The gradients were at a flow rate of 0.3 ml/min, and the column temperatures were kept constant at 25°C. A 2 µL aliquot of each sample was injected.

The ESI source conditions were set as follows: Ion Source Gas1 (Gas1) as 60, Ion Source Gas2 (Gas2) as 60, curtain gas (CUR) as 30, source temperature: 600°C, IonSpray Voltage Floating (ISVF) ± 5500 V. In MS only acquisition, the instrument was set to acquire over the m/z range 60–1,000 Da, and the accumulation time for TOF MS scan was set at 0.20 s/spectra. In auto MS/MS acquisition, the instrument was set to acquire over the m/z range 25–1,000 Da, and the accumulation time for product ion scan was set at 0.05 s/spectra. The product ion scan is acquired using information dependent acquisition (IDA) with high sensitivity mode selected. The parameters were set as follows: the collision energy (CE) was fixed at 35 V with ±15 eV; declustering potential (DP), 60 V (+) and −60 V (−); exclude isotopes within 4 Da, candidate ions to monitor per cycle: 10.

The extracted data were used for metabolite structure identification and subjected to data pre-processing techniques. Subsequently, the data quality was evaluated and analyzed.

### 2.4 Pre-processing of the metabolomics data

The MzXML files were generated from the raw MS data (wiff.scan files). ProteoWizard MSConvert was used for data conversion. Following this, the data were imported into the free XCMS software. The parameters for peak pick up were determined (centWave: m/z, 25 ppm; prefilter, c (10,100); peak width: c (10,60)). The parameters for peak grouping were also set (minfrac: 0.5; bw: 5; mzwid: 0.025). The isotopes and adducts were annotated using the Collection of Algorithms of Metabolite pRofile Annotation (CAMERA). The extracted ion features consisted of variables that were characterized by >50% of the non-zero measurements in at least one of the sets recorded. The accuracy of the m/z values (<25 ppm) and the mass spectroscopy–mass spectroscopy (MS/MS) spectral data were compared with those present in an internal database developed using authentic standards to analyze the metabolites.

### 2.5 Transcriptomics

The heart tissues of the mice belonging to the experimental and control groups were used for the extraction of total RNA. The process of sample extraction was performed using TRIzol. A bioanalyzer (Agilent 2,100) was used to determine the purity and concentration of the extracted RNA. The ribosomal RNA (rRNA) Removal Kit was used for ribosomal RNA removal. The rest of the total RNA samples were subjected to conditions of ionization to break down the samples into fragments that were 200–300 bp long. A random primer consisting of six bases and reverse transcriptase were used to synthesize the first complementary DNA (cDNA) strand. RNA was used as a template during the process. Subsequently, the second strand was generated using the first cDNA strand as the template. This process was followed to generate a specific library. The polymerase chain reaction (PCR) amplification process was used to increase the number of fragments in the library following the process of library construction. Subsequently, based on the library fragment size, the library selection process was conducted (library size: 450 bp). The quality of the libraries was determined using the Agilent 2,100 Bioanalyzer. This same system was also used to test the effective and total library concentrations. The amount of data required for the construction of the library and the effective concentration of the library were analyzed. The mixing of the libraries characterized by different index sequences was based on the results. The mixed libraries were diluted to 2 nM and deformed using alkali to form single-stranded libraries. The libraries were analyzed using the paired-end (PE) sequencing method (Next-Generation Sequencing (NGS); Illumina NovaSeq 6,000 sequencing platform) post the process of extraction and purification of RNA and library construction.

All raw data were filtered to obtain high-quality sequences. These sequences (Clean Data) were aligned with the reference genome. HISAT2 was used for sequence alignment, and this software could be accessed through http://ccb.jhu.edu/software/hisat2/index.shtml. The expression level of each gene was determined based on the alignment results. Subsequently, differential analysis of sample genes was performed using DESeq to identify DEGs satisfying the criteria of |log2FoldChange| > 1 and *p* < 0.05. The ggplots2 package was used to plot the volcano plots for the DEGs.

### 2.6 Metabonomics–transcriptomics combination analysis

The differentially expressed metabolites (DEMs) and gens (DEGs) were extracted. The gens corresponding to the relevant enzymes were also extracted. The relevant data were obtained by analyzing the metabolite information presented in the Kyoto Encyclopedia of Genes and Genomes (KEGG) database. This database can be accessed through the website https://www.kegg.jp/dbget-bin/www_bfind?compound. Finally, DEMs and their associated DEGs were mapped with the corresponding metabolic pathways.

### 2.7 Statistical analysis

All statistical analyses were performed using SPSS 19.0, and the plots were generated using GraphPad Prism 8.0 (statistically significant results: *p* < 0.05). The metabolite-related data were analyzed using ropls (R package). Multiple algorithms were used to realize multivariate data analysis. The orthogonal partial least squares–discriminant analysis (OPLS–DA) and pareto-scaled principal component analysis (PCA) methods were used for data analysis. The 7-fold cross-validation method was used, and response permutation tests were conducted to determine the robustness of the model. For each variable associated with the OPLS–DA model, the variable importance in the projection (VIP) value was calculated. This helped determine the contribution of the variables toward the classification process. The student’s *t*-test was conducted for all metabolites characterized with VIP values > 1. The significance of each metabolite was determined by conducting the tests at the univariate level.

## 3 Results

### 3.1 Sepsis-induced myocardial dysfunction model

**FIGURE 1:**
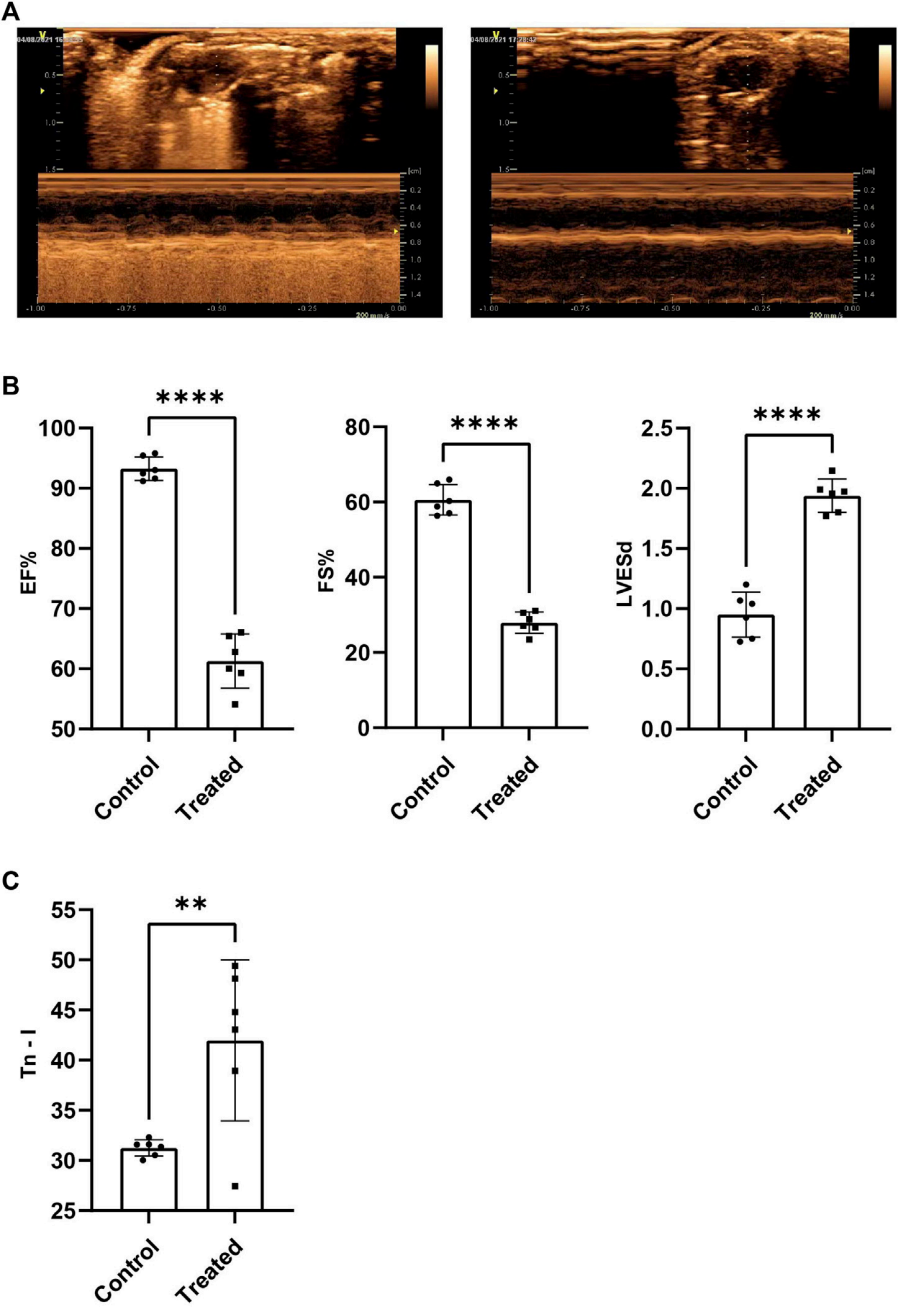
The cardiac function of SIMD mice decreased significantly. **(A)** Representative images of mice heart examined by echocardiography. **(B)**EF%, FS% and LVESd (*n* = 6, *p* < 0.001) **(C)**Tn-I in serum were measured by ELISA assays (*n* = 6, *p* < 0.01).

Results obtained by conducting echocardiography tests suggested that the overall cardiac function of the members of the experimental group, and EF%, FS% recorded for the experimental group were significantly lower than those recorded for the control group (*p* < 0.001) (Figures 1A,B). LVESd recorded for the experimental group is significantly higher than the control group (*p* < 0.001) (Figure 1B). It was also observed that the circulating Tn-I level in the experimental group was significantly higher than the Tn-I level recorded for the control group (*p* < 0.05) (Figure 1C). These indicated the successful establishment of the SIMD model.

### 3.2 Metabolomics validation of the model

**FIGURE 2:**
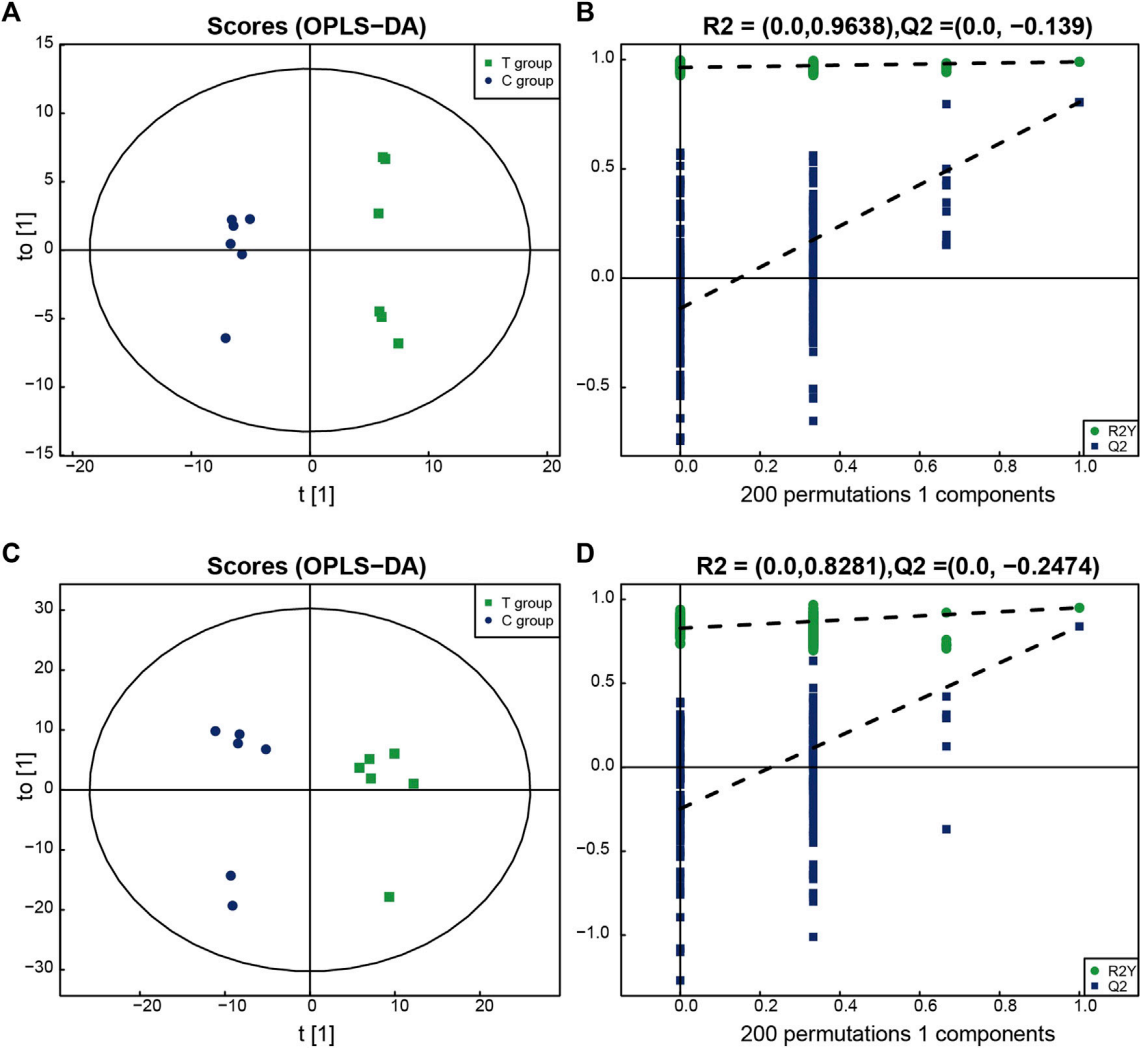
The multidimensional results in positive and negative ionization modes are shown in this figure. **(A,B)** OPLS-DA score plot and OPLS-DA validation plot intercepts in positive ionization modes: the Treated group vs. the Control group. R2Y = (0.0, 0.9638), Q2 = (0.0,−0.139). **(C,D)** OPLS-DA score plot and OPLS-DA validation plot intercepts in negative ionization modes: the Treated group vs. the Control group. R2Y = (0.0, 0.8281), Q2 = (0.0,−0.2474).

All the identified metabolites were analyzed using a multi-dimensional statistical analysis method. The OPLS–DA permutation test plot and the OPLS–DA score plot generated under both the positive and negative ion modes are shown in the Figure 2. It was observed that the model could be used to differentiate between one group of samples from the other, and overfitting could be avoided. This indicated the good robustness of the model.

### 3.3 Identification of differentially expressed metabolites

**FIGURE 3:**
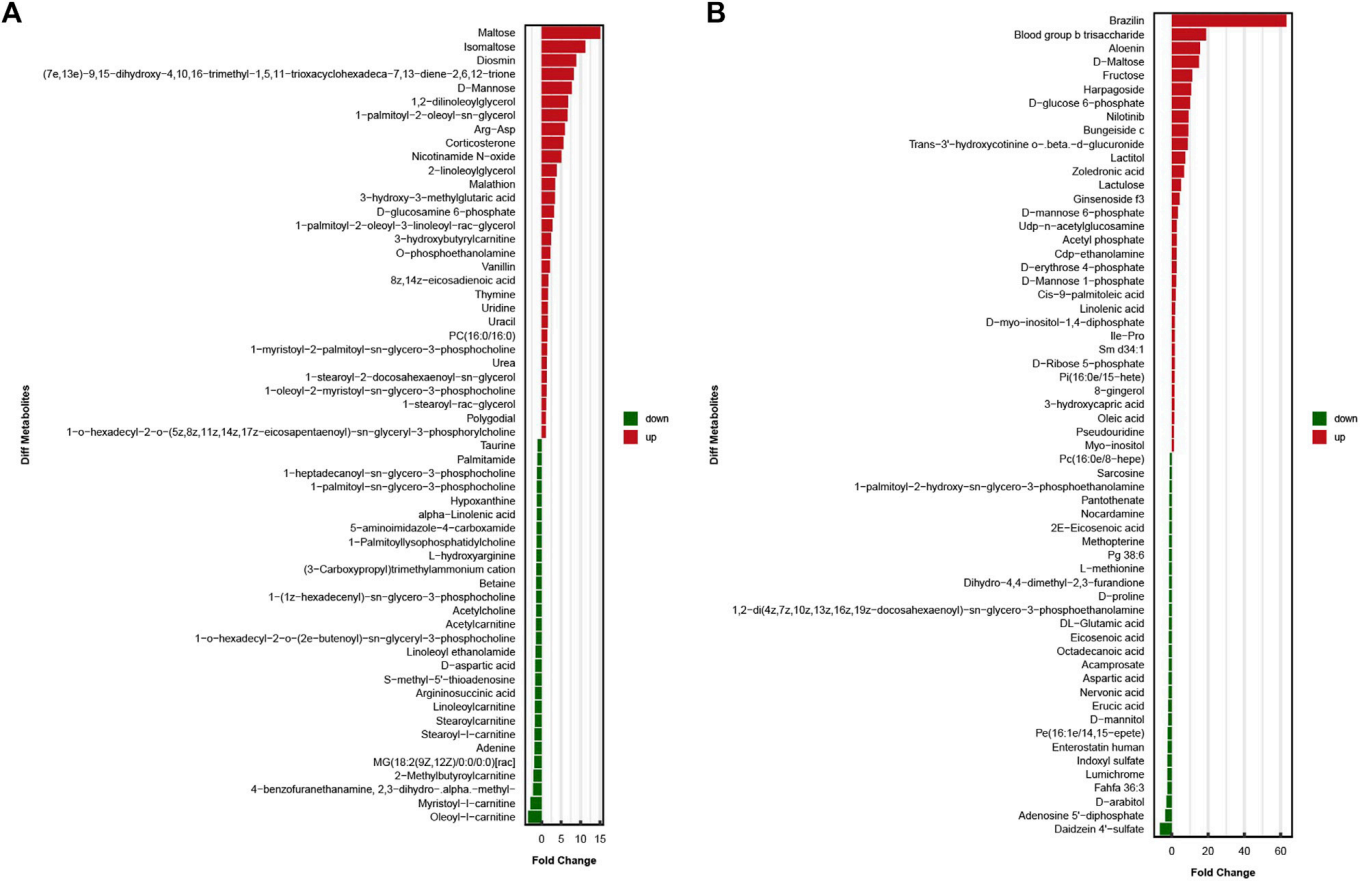
In two ionization modes, one-dimensional, and multi-dimensional analysis results of differential metabolites. **(A)**significant differences in metabolite expression in positive ionization modes. **(B)**significant differences in metabolite expression in negative ionization modes.

The results obtained under the positive and negative ion modes were combined, and a total of 1,027 metabolites were identified. Of all these samples, 390 metabolites were identified under the positive ion mode, and 637 metabolites were identified under the negative ion mode. Univariate and multi-dimensional analyses methods were used to screen 118 DEMs (criteria for OPLS–DA: VIP >1; *p* < 0.05). Among these, 58 significant DEMs were identified under the cationic mode, and 60 significant DEMs were identified under the anionic mode. The results obtained under these two modes are presented in the Figure 3.

### 3.4 Transcriptomics analysis of sepsis-induced myocardial dysfunction

**FIGURE 4:**
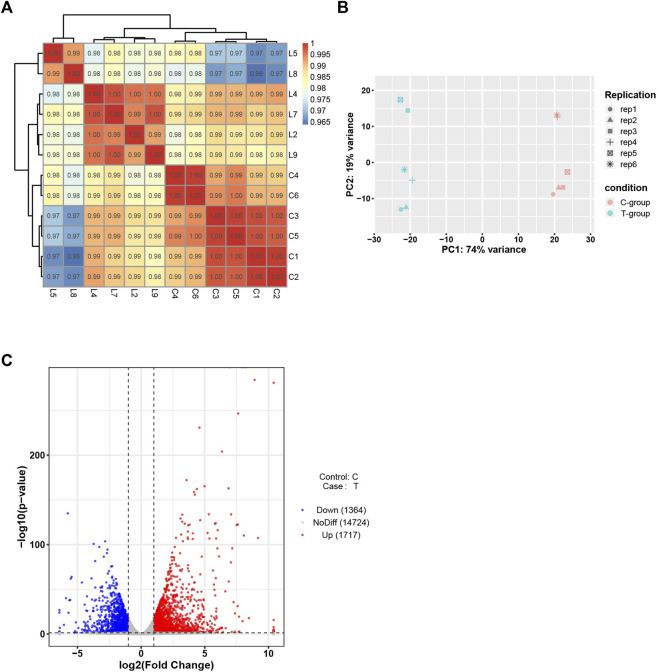
Bioinformatics analysis of RNA-seq data. **(A)** Sample correlation test. **(B)**PCA of mRNAs. **(C)** Volcano plot of mRNAs. C-group the control group (*n* = 6). T-group the SIMD group (*n* = 6).

High-throughput transcriptome analysis of the heart tissues in the experimental and control groups was performed. The correlation coefficients between the samples ranged from 0.8 to 1, indicating an extremely strong correlation. PCA results indicated high intra-group similarity between the samples in the experimental and control groups. Of the 3,081 DEGs recorded, down-regulation was observed for 1,364 DEGs, and up-regulation was observed for 1717 DEGs (Figure 4).

### 3.5 Metabolomics–transcriptomics combination analysis

**FIGURE 5:**
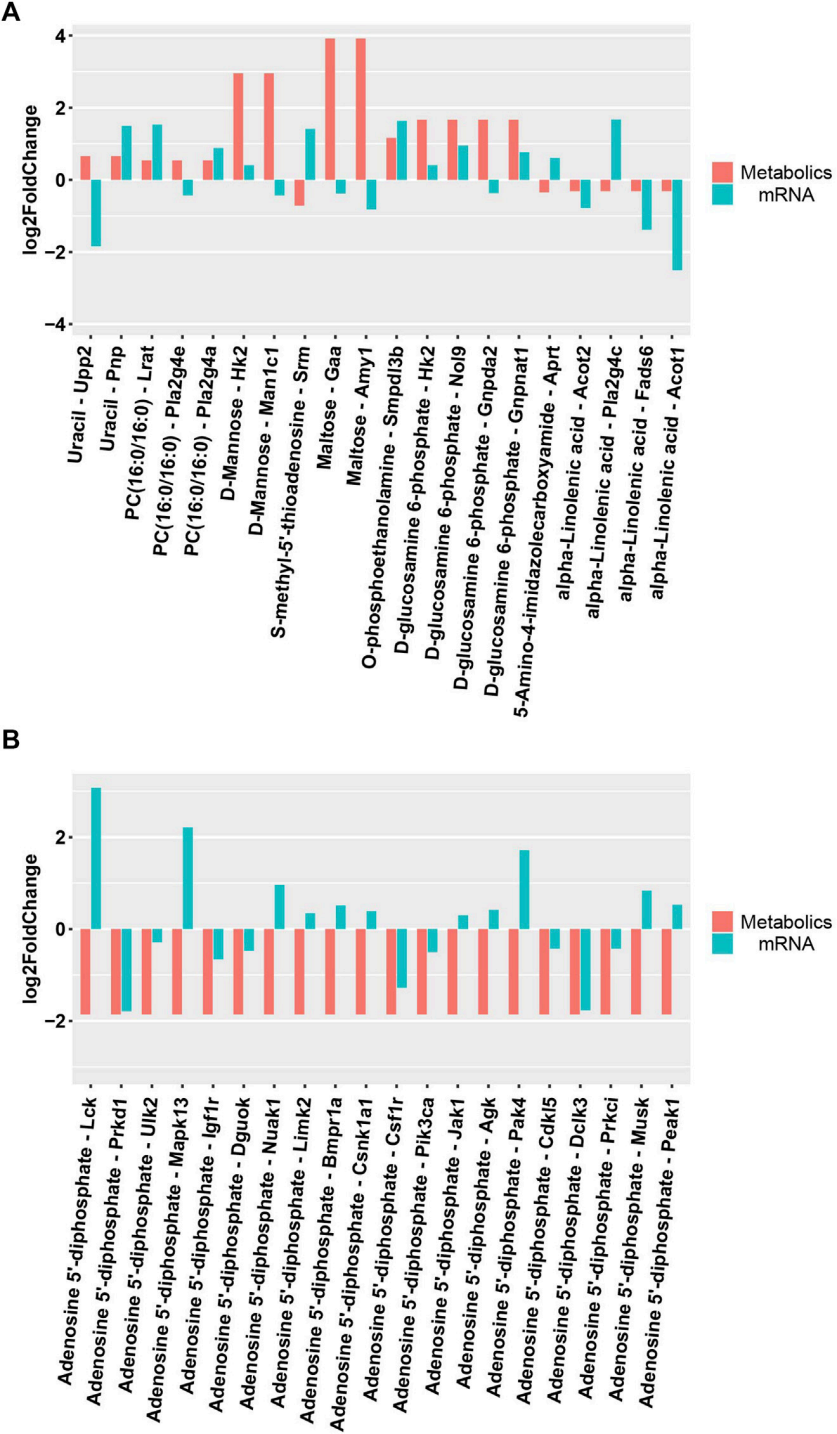
Differential expression of metabolites and related transcripts. **(A)**Differential expression of metabolites and related transcripts in positive ionization modes. **(B)** Differential expression of metabolites and related transcripts in negative ionization modes.

**FIGURE 6:**
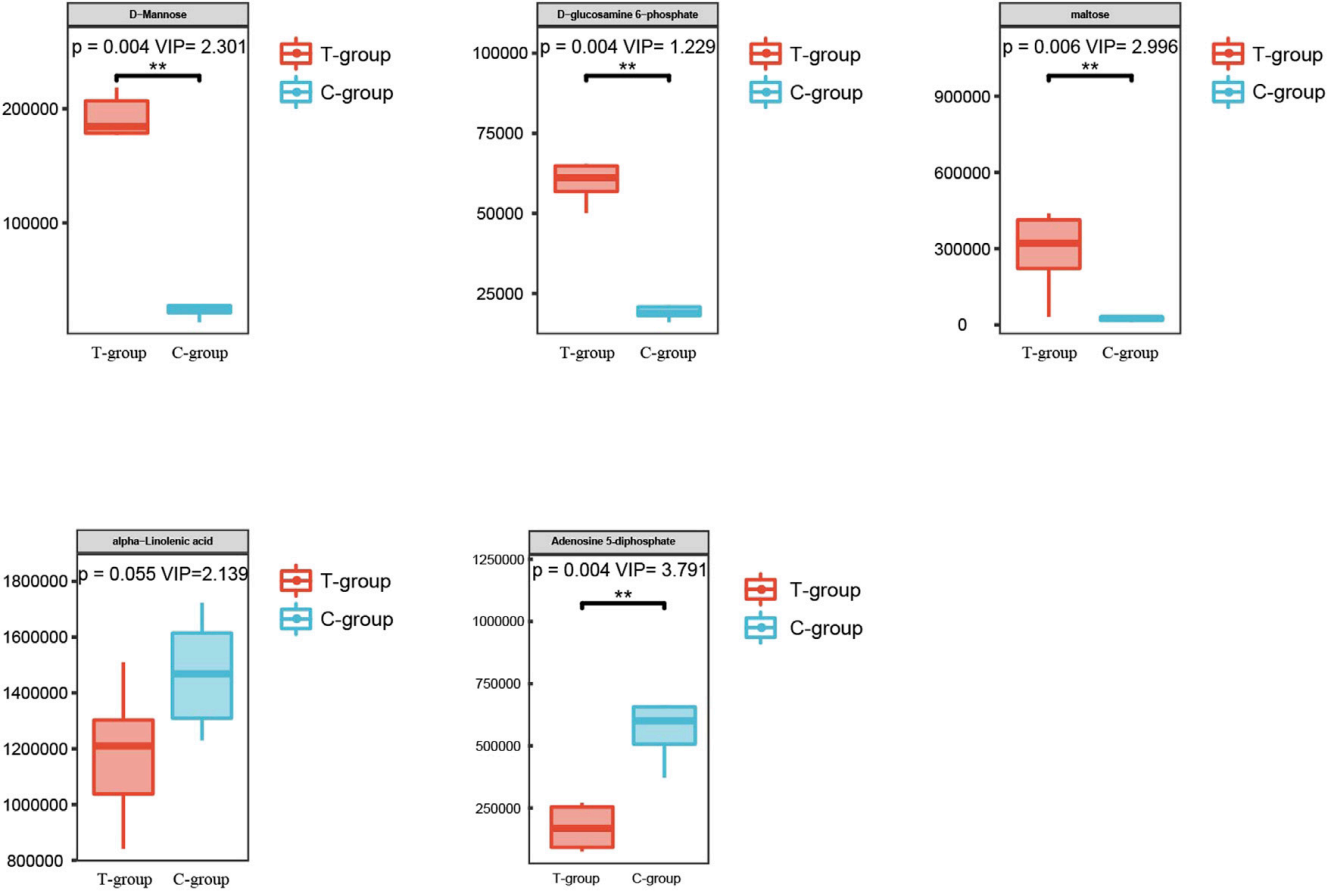
Metabolites with significant differences between the two groups.

DEMs obtained under the negative and positive ion modes and the transcriptome data were subjected to conditions of the metabolomics–transcriptomics combination analysis method. The change in the fold of the top 20 DEM–DEG pairs is shown in the Figure 5. Finally, multiple common metabolites were identified by analyzing the mice in both groups. The common metabolites were identified to be d-mannose, d-glucosamine 6-phosphate, maltose, alpha-linolenic acid, and adenosine 5′-diphosphate (Figure 6).

### 3.6 Differentially expressed metabolites and differentially expressed genes: Analysis of the kyoto encyclopedia of genes and genomes pathway

The DEMs and DEGs were mapped simultaneously to the KEGG pathway database to identify the common pathways associated with the DEMs and DEGs (Supplementary Tables S1–S9). The results are presented in the Table 1.

**TABLE 1:** Pathways of metabolites and related transcripts.

DEMs	DEGs	Pathway
d-Mannose	Hk2	Fructose and mannose metabolism
d-Glucosamine 6-phosphate	Gnpda2Hk2; Gnpnat1	Amino sugar and nucleotide sugar metabolism
Maltose	Gaa	Starch and sucrose metabolism
	Amy1	Carbohydrate digestion and absorption
Alpha-Linolenic acid	Acot1	Biosynthesis of unsaturated fatty acids
Adenosine 5′-diphosphate	Prkci	Platelet activation
	Igf1r; Pik3ca	AMPK signaling pathway
	Dguok	Purine metabolism

## 4 Discussion

Metabolomics allows for a more precise exploration of disease diagnosis and pathogenesis. The metabolomics–transcriptomics combination analysis method used helped us to identify significant DEMs between the two groups, including d-mannose, maltose, d-glucose 6-phosphate, alpha-linolenic acid, and adenosine 5′-diphosphate. Additionally, metabolite-related metabolic pathways were also investigated.

d-mannose, a common monosaccharide, is a digestive product of polysaccharides and glycoproteins. However, the amount of mannose present in the daily diet is significantly small. Hexokinase converts mannose to mannose-6-phosphate, which is then converted to fructose 6-phosphate by mannose phosphate isomerase. This eventually participates in the glycolytic pathway to produce lactic acid, glucose, and pentose (Wood and Cahill, 1963; Ganda et al., 1979). Elevated lactate levels indicate cellular dysfunction in patients with sepsis. Hyperlactataemia is a sign of severe sepsis and results in high mortality (Singer et al., 2016). Mannan-binding lectin (MBL) is a crucial complement component in the human body and is an important part of the processes associated with innate immunity. Infection caused by pathogenic microorganisms induces the secretion of MBL, which specifically recognizes and binds to mannose on the surface of microorganisms. This triggers complement activation and mediates the process of generation of inflammatory response (Fujita, 2002). It has been reported that in the sera of individuals with sepsis attributable to Gram-negative bacterial infections, MBL recognizes and binds to mannose on LPS to activate the complementary MBL pathway and initiate the body’s innate immunity to participate in the inflammatory response (Fujita, 2002). This results in a significant reduction in the MBL levels. The results reported herein reveal that the mannose levels in the heart tissues of mice with LPS-induced SIMD were significantly higher than the mannose levels recorded for the control group. Additionally, the expression level of Hk2, a gene that mediates the process of d-mannose metabolism, was significantly high. A large amount of d-mannose was deposited in cardiac tissues, and this activated MBL to trigger innate immune responses and induce an inflammatory response.

d-glucosamine 6-phosphate, a type of glucosamine, is an important energy source for many bacteria present in the body. It is also an important component of bacterial cell walls (Matsuura, 2013). Moreover, d-glucosamine 6-phosphate is also associated with the virulence of some bacteria (Kawada-Matsuo et al., 2016).

Maltose is a disaccharide that is produced in the body during starch catabolism. It can be metabolized to form two glucose molecules. Researchers have previously used magnetic resonance imaging-based metabolomics techniques to study conditions of sepsis. The results revealed that the maltose content in the metabolites of patients with sepsis was significantly lower than the maltose contents of patients not suffering from sepsis. However, no such changes were observed in the sham-operated and control groups (Bakalov et al., 2016). This suggested that the significant reduction in the maltose content was associated with the chronic depletion of the long-term inflammatory response. We used an early-state 6 h animal model to conduct the studies. The experimental results suggested a significant increase in the maltose content. However, whether the maltose content changes as sepsis progresses needs to be further investigated.

Alpha-linolenic acid (ALA) is a type of omega-3 essential fatty acid. It is a polyunsaturated fatty acid with three double bonds. It has been previously reported that ALA and its metabolites significantly inhibit the generation of LPS-induced inflammatory response, and their action results in a decrease in the rate of cellular reactive oxygen species (ROS) and NO production. These could also inhibit the expression of iNOS and TNF-α in cells and reduce the mortality in mice suffering from endotoxin-mediated septic shock (Kumar et al., 2016).

Mitochondrial dysfunction is an adverse mechanism associated with the cardiac dysfunction observed in patients with sepsis (Ravikumar et al., 2021). It results in the inability of the body to synthesize sufficient amounts of adenosine triphosphate (ATP) to provide energy for the heart (Wasyluk et al., 2021). Insufficient ATP synthesis also results in a reduction in the adenosine diphosphate (ADP) content in cardiac tissues. This result agrees with the results reported herein. It was also observed that the amount of adenosine 5′-diphosphate in the heart tissues of mice in the experimental group was significantly lower than the content of adenosine 5′-diphosphate in the heart tissues of mice belonging to the control group.

DEMs and DEGs were linked to mannose metabolism, aminoglycan metabolism, starch metabolism, unsaturated fatty acid biosynthesis, platelet activation, purine metabolism, and AMP-activated protein kinase (AMPK) signaling pathways. AMPK significantly affects the process of cellular energy homeostasis (Carling et al., 2011). Stressors such as hypoglycemia, hypoxia, and ischemia that remarkably deplete ATP can activate this pathway (Canto and Auwerx, 2010; Hardie, 2011; Mihaylova and Shaw, 2011), which positively regulates the signaling pathways that replenish cellular ATP supply.

There are some limitations to this study. Although LPS is an important myocardial inhibitory factor, the predisposing factors for cardiac dysfunction are not limited to Gram-negative bacteria-induced sepsis. Therefore, we will further explore the metabolic alterations and pathogenic mechanisms associated with Gram-positive bacteria-induced SIMD in the future.

## 5 Conclusion

In summary, significant changes in metabolites occur in the cardiac tissues of patients suffering from SIMD. These changes are primarily associated with mannose metabolism, aminoglycan metabolism, starch metabolism, unsaturated fatty acid biosynthesis, platelet activation, purine metabolism, and AMPK signaling pathways. The problems associated with the aberrant metabolic events can be addressed to help improve the prognoses of patients with SIMD and provide new insights into the processes associated with diagnosis and disease management.

## Data Availability

The authors acknowledge that the data presented in this study must be deposited and made publicly available in an acceptable repository, prior to publication. Frontiers cannot accept a manuscript that does not adhere to our open data policies.

## Abbreviations

SIMD, Sepsis-induced myocardial dysfunction; LPS, lipopolysaccharide; KEGG, Kyoto Encyclopedia of Genes and Genomes; DEMs, differentially expressed metabolites; AMPK, AMP-activated protein kinase; DEGs, differentially expressed genes; LVEF, left ventricular ejection fraction; LVEDd, left ventricular end-diastolic dimension; LVESd, left ventricular end-systolic dimension; LVFS, left ventricular fractional shortening; NGS, Next-Generation Sequencing; OPLS-DA, orthogonal partial least squares–discriminant analysis; PCA, principal component analysis; ALA, Alpha-linolenic acid.
