# Supplementary material for: Analysis of metabolic disturbances attributable to sepsis-induced myocardial dysfunction using metabolomics and transcriptomics techniques
Source: Front Mol Biosci. 2022 Aug 15;9:967397. doi: 10.3389/fmolb.2022.967397 (PMC9421372; doi:10.3389/fmolb.2022.967397)
Supplement: Supplementary file 3 [file Table1.docx]

Table 1: Pathways of metabolites and related transcripts.

| DEMs | DEGs | Pathway |
| --- | --- | --- |
| D-Mannose | Hk2 | Fructose and mannose metabolism |
| D-Glucosamine 6-phosphate | Gnpda2Hk2; Gnpnat1 | Amino sugar and nucleotide sugar metabolism |
| Maltose | Gaa  Amy1 | Starch and sucrose metabolism  Carbohydrate digestion and absorption |
| Alpha-Linolenic acid | Acot1 | Biosynthesis of unsaturated fatty acids |
| Adenosine 5'-diphosphate | Prkci  Igf1r; Pik3ca  Dguok | Platelet activation  AMPK signaling pathway  Purine metabolism |
